# The Development and Pre-Clinical Anti-Inflammatory Efficacy of a New Transdermal Ureasil–Polyether Hybrid Matrix Loaded with Flavonoid-Rich *Annona muricata* Leaf Extract

**DOI:** 10.3390/pharmaceutics16081097

**Published:** 2024-08-21

**Authors:** Camila Beatriz Barros Araújo, José de Oliveira Alves Júnior, Mariana Rillo Sato, Kammila Martins Nicolau Costa, Jéssica Roberta Lima, Bolívar Ponciano Goulart de Lima Damasceno, Francisco José Batista de Lima Junior, Bruna Galdorfini Chiari Andréo, Vanda Lucia dos Santos, João Augusto Oshiro-Junior

**Affiliations:** 1 Pharmaceutical Sciences Postgraduate Center for Biological and Health Sciences, Paraiba State University, Av. Juvêncio Arruda, S/N, Campina Grande 58429-600, Brazil; camilabeatriz2300@gmail.com (C.B.B.A.); mari_sato_@hotmail.com (M.R.S.); bolivarpgld@servidor.uepb.edu.br (B.P.G.d.L.D.); vandalsantos@servidor.uepb.edu.br (V.L.d.S.); 2 Department of Pharmacy, Center for Biological and Health Sciences, Paraiba State University, Av. Juvêncio Arruda, S/N, Campina Grande 58429-500, Brazil; oliveiraj.alves@yahoo.com; 3 Postgraduate Program in Development and Technological Innovation in Medicines, Federal University of Paraíba, Campus I, Lot. Cidade Universitária, S/N, João Pessoa 58051-900, Brazil; kammilamartiins@hotmail.com; 4 Department of Pharmacy, University of Araraquara (UNIARA), Rua Carlos Gomes, 1338—Centro Araraquara, São Paulo 14801-340, Brazil; jessicageane@yahoo.com.br (J.R.L.); brunagchiari@yahoo.com.br (B.G.C.A.); 5 Department of Pharmacy, Unifacisa University Center, Rua Manoel Cardoso Palhano, 124-152—Itararé, Campina Grande 58408-326, Brazil; francisco.lima@maisunifacisa.com.br

**Keywords:** transdermal anti-inflammatory therapy, ureasil–polyether, Annonaceae family, sol-gel process, new therapeutic systems

## Abstract

This study aimed to develop a novel ureasil–polyether transdermal hybrid matrix (U-PEO) loaded with *Annona muricata* concentrated extract (AMCE), which exhibits potent anti-inflammatory activity. The extract was obtained by maceration, a method that allowed for the extraction of a high concentration of flavonoids (39.27 mg/g of extract). In vivo tests demonstrated that 10 mg/kg of AMCE inhibited inflammation for 6 h. The physicochemical characterization of U-PEO with AMCE was conducted via a thermogravimetric analysis (TGA), while its surface was recorded using atomic force microscopy (AFM). The in vitro macroscopic swelling and release tests demonstrated the hydrophilic profile of the material and the percentage of AMCE released. The TGA results demonstrated that the system exhibited physical compatibility due to the thermal stability of U-PEO. Additionally, the AFM analysis revealed a rough and porous surface, with a particular emphasis on the system with AMCE. The release resulted in the liberation of 23.72% of AMCE within 24 h. Finally, the preclinical tests demonstrated that U-PEO with AMCE was also capable of effectively inhibiting inflammation for 6 h, a duration comparable to that of a commercial formulation. The results permit the advancement of the study towards the development of a transdermal system, thereby rendering its application in clinical studies feasible.

## 1. Introduction

Inflammation is a rapid and self-limiting immune protection mechanism triggered in response to tissue damage or foreign substances in the body. Its process is associated with the coordinated action of immune biochemical reactions, cell migration or recruitment, and the release of pro-oxidant factors that produce reactive oxygen species (ROS) or reactive nitrogen species (RNS) [1]. In this context, when this process is dysregulated, it can result in the onset of acute or chronic disorders, which may even lead to functional impairment [2].

The conventional treatment involves the administration of costly synthetic drugs that are frequently associated with significant adverse effects, including glucocorticoids, statins, non-steroidal anti-inflammatory drugs (NSAIDs), and even metformin [3,4]. As an alternative, research involving the exploitation of plant active pharmaceutical ingredients (PAPI) has become more frequent given that these products are sources of bioactive compounds with various known and consolidated therapeutic activities [2].

In this context, the PAPI of *Annona muricata* (AM) extract has emerged as a promising candidate for the treatment of acute or chronic inflammatory diseases due to its pronounced content of phenolic constituents, such as flavonoids, which are present throughout the plant’s structure, including the leaves [5]. Some studies have associated this effect with the suppression of tumor necrosis factor-alpha (TNF-α) and Interleukin 1 beta (IL-1β) as well as the modulation of nitric oxide (NO) [6]. It is postulated that the antioxidant action occurs through the positive regulation of superoxide dismutase-1 (SOD-1) and nuclear factor erythroid-2-related factor (Nrf-2), which favors the reduction of reactive oxygen species activity [6].

In addition, the route of administration is also a factor that must be taken into account. The treatment of inflammatory diseases typically employs oral and intramuscular routes. However, while the former is associated with low bioavailability, the latter is linked to pain, skin irritation, and the rapid metabolization of the active ingredient, which can impede patient adherence to treatment [7,8]. In light of these considerations, reservoir-type transdermal therapeutic systems (TDDSs) may be employed as an alternative to address these challenges. A TDDS facilitates the transdermal delivery of drugs, requiring only a reservoir matrix with suitable mechanical properties and the capacity to trap the active ingredient.

In this sense, ureasil–polyether hybrid materials stand out for their mechanical strength, biocompatibility, easy handling, high flexibility, and ability to trap hydrophilic and lipophilic substances, as they combine organic and inorganic characteristics [9,10,11,12]. Their efficiency in loading drugs to saturation makes them excellent choices in developing reservoir matrices that can be used in developing transdermal therapeutic systems. Moreover, these materials are produced via the well-known sol-gel process, which involves the transformation from a solution (sol) to a solid matrix (gel). This method offers several key benefits, including the ability to create diverse pharmaceutical forms using low energy, a favorable cost–benefit ratio, and the possibility of controlling the chemical composition and final physical properties of the matrix [13].

In this context, the present study focused on the development of a ureasil–poly(ethylene oxide) (U-PEO) reservoir matrix for the modified release of AM extract and to preclinically investigate its anti-inflammatory potential for future use as a final transdermal drug delivery system (TDDS) in clinical studies.

## 2. Materials and Methods

### 2.1. Materials

*O*,*O*′-Bis(2-aminopropyl) polypropylene glycol-block-polyethylene glycol-block-polypropylene glycol (PEO); 3-(triethoxysilyl)propylisocyanate (IsoTrEOS); 2,2-diphenyl-1-picrylhydrazyl (DPPH) (purity ≈ 90%); and quercetin hydrate (purity ≥ 95%) were obtained from Sigma-Aldrich^®^ (São Paulo, Brazil). Flexive^®^ (*Symphytum officinale* L.) from P&G Health Austria (Spittal an der Drau, Austria) (Lot: 1158C60104) and to Indomethacin^®^ from Sigma-Aldrich (St. Louis, MO, USA) (Lot: BCBC9386). The phosphate buffered saline (PBS) (Dulbecco A) pH 7.2 belonged to Oxoid Ltd. (Basingstoke, UK), and the aluminum chloride (AlCl_3_·6H_2_O) was from Dinâmica Química Contemporânea Ltd.a. (Indaiatuba, Brazil). HCl at 2 M (purity ≥ 36.5%) was obtained from Qhemis^®^ (São Paulo, Brazil). The human hepatoma cell line was purchased from the cell line bank (ATCC Cell Bank, London, UK), Dulbecco’s Modified Eagle Medium (DMEM) (Sigma-Aldrich, UK) supplemented with 1% Penicillin-streptomycin and 10% fetal bovine serum (FBS) (Sigma-Aldrich, UK). Thiazolyl blue tetrazolium bromide (MTT) was obtained from Sigma-Aldrich (UK). Finally, the solvents absolute ethanol (purity 99.5%) (Dinâmica Química Contemporânea Ltd., Indaiatuba, Brazil), methyl alcohol ethanol (purity 99.9%), and isopropyl alcohol (purity 99.5%) (REATEC Ltd., São Paulo, Brazil) were of analytical grade.

### 2.2. Plant Material

The leaves of AM were collected in the municipality of Puxinanã in the semi-arid region of the state of Paraiba on 4 February 2022 at 09:00 (7°10′04.7″ S; 35°58′50.1″ W). A voucher specimen was deposited in the Manuel de Arruda Câmara Herbarium of the State University of Paraíba (UEPB) under the number 001966 as well as registered in the National System for the Management of Genetic Heritage and Associated Traditional Knowledge (SISGEN) under the registration AC28672.

The leaves were washed with a 2% sodium hypochlorite solution *v*/*v* and then dried in a forced air circulation oven (TECNAL model TE-394/2 MP, São Paulo, Brazil) at 40 °C until the mass of the material was stable. Finally, the leaves were pulverized in a knife mill (SOLAB model SL-30, São Paulo, Brazil) with a 10-mesh mesh. The standardization tests and analysis of the dried leaf powder (DLP) were carried out according to the technique proposed by the Brazilian Pharmacopoeia 6th edition [14].

### 2.3. Obtaining Concentrated Extracts from the Dried Leaves of A. muricata

Preliminarily, three different techniques (maceration, turbolysis, and ultrasound) were employed to produce batches of 1 L of the extract, with the objective of identifying the method that would afford the highest concentration of flavonoids, expressed as quercetin equivalents (QE), per gram of concentrated extract. For all the methods, three proportions of hydroalcoholic solvent (30, 50, and 70% *v*/*v*) based on water and absolute ethanol and three concentrations of DLP (2, 6, and 10%) were tested.

Maceration was conducted for 72 h in a sealed graduated bottle at room temperature (25 ± 2 °C), in the absence of light, and without constant stirring. At 24 h intervals, the bottle was manually shaken for a few seconds. Subsequently, the samples were analyzed at the end of the aforementioned maceration period. The ultrasonic and turbolysis methods were conducted using a 2 L glass beaker. For the former, the samples were placed in an ultrasonic cleaner (UNIQUE model USC 1800A, São Paulo, Brazil) for 30 min at a constant power of 132 W and a frequency of 40 kHz. In the second method, the samples were subjected to agitation in an ultraturrax (TECNAL model TE-102 TURRATEC, São Paulo, Brazil) at 10,000 rpm for a total of four cycles, each consisting of five minutes of agitation with a one-minute rest interval between cycles. The total duration of the second method was thus 20 min.

Once the total flavonoid content (Section 2.3.1) was determined and the method and proportions of ingredients that provide the highest flavonoid content per QE were selected, 5 L of AM concentrated extract (AMCE) was prepared. In turn, the concentrate was filtered, and the solvent was rotary evaporated under reduced pressure in a rotary evaporator (IKA^®^ RV8, Staufen, Germany) at 75 °C. The mixture was transferred to 20 × 20 cm Petri dishes and subjected to a 3-day incubation at 50 °C in an oven (ETHIK TECHNOLOGY model 402-4D, São Paulo, Brazil).

#### 2.3.1. Phytochemical Prospecting: Determination of Total Flavonoid Content

The analytical curve was constructed using concentrations of 6 to 28 µg/mL of quercetin and analyzed in a UV-VIS spectrophotometer (UVmini-1240—Shimadzu, Kyoto, Japan) to read the absorbance at 415 nm. The plant extract samples were read in the presence of aluminum chloride solution (AlCl_3_), and the absorbance values found were related to the concentration of flavonoids in the solution through a mathematical relationship with the calibration curve.

An aliquot of 1.5 mL of a methanolic solution of AlCl_3_ 2% *w*/*v* was poured into test tubes, followed by 1.5 mL of the methanolic solution of the extract. The tubes were left to stand for 10 min at room temperature (25 °C). Measurements were taken at a wavelength of 415 nm. The concentration of the extract was corrected experimentally to be within the linear range of spectral absorbance obtained from the calibration curve. The absorbance result was corrected by reading a sample containing 1.5 mL of the extract solution and 1.5 mL of methanol and subtracting the absorbance values of the tubes without reagents from those containing reagents. The values are expressed in QE.

#### 2.3.2. Cytotoxicity Assay in Human Hepatoma Cell Line (HepG2)

The HepG2 cells were cultivated in a Dulbecco’s Modified Eagle Medium (DMEM) culture medium and maintained in an incubator with 5% CO_2_ and a temperature of 37 °C until the cell monolayer reached confluence. Subsequently, the cells were dissociated using trypsin and homogenized with culture medium plus 10% fetal bovine serum. For the cell viability and cytotoxicity tests using 3-[4,5-dimethylthiazol-2-yl]-2,5-diphenyltetrazolium bromide (MTT), a suspension of 2.5 × 10^5^ cells per well of the strain was utilized.

The cells were cultured in 96-well plates for 24 h. Different concentrations of AMCE (0.05 to 212 µg/mL) were solubilized in DMEM and added to the wells. After 24 h, the medium was discarded, and 100 μL of MTT (1 mg/mL) in PBS was added to each well. The cells in the microplate were incubated at 37 °C and protected from light for approximately 3 h until the presence of violet formazan crystals was observed. To solubilize the crystals, 100 μL of absolute isopropyl alcohol was added to each well, and the absorbance was read spectrophotometrically at a wavelength of 595 nm using a plate reader (FLUOstar, BMG Labtech, Ortenberg, Germany). The experiments were conducted in triplicate, and the IC_50_ was calculated using the equation obtained from the linear regression of the results versus the AMCE concentration. The percentage of live cells was calculated in relation to the negative control, and the data were analyzed using Origin^®^ 8.5 software (OriginLab Corporation, Northampton, MA, USA).

#### 2.3.3. Evaluation of Antioxidant Activity Using the DPPH Radical Scavenging Test

The antioxidant capacity of AMCE was determined using the 1,1-diphenyl-2-picrylhydrazyl (DPPH) radical scavenging assay. Based on the adapted technique of Leite et al. [15], different concentrations of the sample (5–10–20–30–40–50 µg/mL) were prepared in absolute HPLC methyl alcohol, then 1.5 mL of these was added to 1.5 mL of 0.20 mM DPPH methanolic solution.

The solutions were stored away from light for 30 min and then read in UV-VIS at 515 nm. In addition, the solution of 1.5 mL of DPPH added to 1.5 mL of methanol was used as a control (Leite et al., 2019). Finally, the average of the absorbances was used to calculate the percentage of inhibition and capture of the DPPH radical, stipulated by Equation (1).

(1) $$\%\;\mathrm{DPPH}\;\mathrm{inhibition}=\frac{\left({ABS}_{CONTROL}-{ABS}_{SAMPLE}\right)}{{ABS}_{CONTROL}}\;\times \;100$$

considering ${ABS}_{CONTROL}$ as the absorbance of DPPH at 515 nm without the addition of any samples as well as ${ABS}_{SAMPLE}$ the absorbance of DPPH at 515 nm in the presence of the samples. In addition, the IC_50_ was calculated using the equation obtained from the linear regression of the results versus the AMCE concentration.

### 2.4. Synthesis of Ureasil–Polyether Hybrid Matrices

The initial synthesis of the hybrid precursor (PEO) was performed through the sol-gel method, employing a functionalization reaction between the polymer poly(ethylene oxide) (H_2_N-PEO-NH_2_) (Mw = 500 g/mol) and the modified alkoxide 3-isocinatopropyltriethoxysilane (IsOTrEOS) in a 1:2 molar ratio (polymer/alkoxide). The mixture was maintained under reflux and stirred for 24 h at 76 °C. Thereafter, the ethanol was removed under vacuum at 78 °C and 267 mbar using a rotaevaporator [16].

Subsequently, the hybrid matrices were obtained by hydrolysis and condensation reactions by dissolving 0.750 g of the precursor in 500 µL of ethanol, 100 µL of water, and 50 µL of the acid catalyst (HCl at 2 M), homogenized in that order at 300 rpm for 2 min. AMCE was dissolved in the ethanol/water solution and incorporated into the PEO in different proportions of 1% *w*/*v* (7.5 mg), 3% *w*/*v* (22.5 mg), 6% *w*/*v* (45 mg), 10% *w*/*v* (75 mg), and 20% *w*/*v* (150 mg) related to the precursor mass to identify the maximum possible trapping concentration. The process was concluded with the addition of the catalyst. Finally, the hybrid matrices were kept dry in a desiccator at room temperature (25 ± 2 °C).

### 2.5. Physicochemical Characterization of Hybrid Matrices

#### 2.5.1. Thermogravimetric Analysis (TGA)

The thermogravimetric curves of the DLP, AMCE, PEO, and U-PEO hybrid matrix loaded or not loaded (isolated) with AMCE were generated by a simultaneous thermal analyzer (Shimadzu™—DTG-60, Shimadzu, Kyoto, Japan) under a nitrogen atmosphere with a flow rate of 100 mL/min and heating ratio (*β*) of 10 °C/min. To do this, about 6 mg of each ingredient was weighed into an open alumina crucible and exposed to a temperature range of 30 to 900 °C. The program TA-60WS version 2.21 (Shimadzu, Kyoto, Japan) was used to visualize the TGA curves as well as their derivative curves.

#### 2.5.2. Atomic Force Microscopy (AFM)

The topographic profile of U-PEO was evaluated using the atomic force microscopy (AFM) technique. For this purpose, the atomic force microscope (Bruker Multimode III, Dimension Icon model, Santa Barbara, CA, USA) was employed in intermittent contact mode to record the images. The hybrid matrices were positioned on the equipment, and their surface was analyzed by a probe or cantilever under a scanning area of 50 × 50 μm [17,18]. The topographic images were processed for relief and porosity using the NanotecWSxM software, version 5.0 [18].

#### 2.5.3. Macroscopic Swelling Test

The loaded or unloaded hybrid matrix of AMCE was inserted into individual beakers containing 500 mL of phosphate buffer pH 7.2 at a temperature of 36.5 ± 0.5 °C and shaken at 300 rpm. At predetermined intervals (0, 0.25, 0.5, 1, 2, 3, 4, 6 h), the U-PEO were removed, dried, and weighed on an analytical balance, as previously validated by Barros et al. [11]. The water content absorbed during the assay was monitored using the swelling ratio (S_W_) according to Equation (2).

%S_W_ = (W1 − W2) × 100 (2)

considering W1 and W2 as the masses of the swollen and non-swollen U-PEO hybrid matrices, respectively.

### 2.6. In Vitro A. muricata Release Assay

The release test was conducted in a paddle dissolution apparatus that had been previously calibrated (Model 299-3, Ethik Instruments, São Paulo, Brazil). To achieve this, the U-PEO matrix containing AMCE (n = 6) was immersed in 900 mL of phosphate-buffered receptor medium with a pH of 7.2 and kept stirring at 50 rpm and 36.5 ± 0.5 °C. At predetermined times of 0.25, 0.5, 0.75, 1, 2, 4, 6, 8, 10, 12, and 24 h, 1.5 mL aliquots of the receiving medium were removed to determine the concentration of AMCE as a function of time using a spectrophotometer at 415 nm (Section 2.3.1). The same volume of the removed aliquot was replaced, ensuring that the receiving medium remained under identical conditions throughout the process.

The data obtained were employed to ascertain the cumulative concentration of AMCE, expressed in QE, released from the hybrid matrix over time. The release kinetics were then evaluated by fitting the results in percentage to different kinetic models, such as zero order, first order, Weibull, and Hixon and Crowell models, using SigmaPlot 10.0 software (Systat Software Inc., San Jose, CA, USA).

### 2.7. In Vivo Assays

#### 2.7.1. Animals

The in vivo test was conducted at the Pharmacological Testing Laboratory of the State University of Paraíba (UEPB), registered under No. 027/2022. The care and handling of the animals were performed following the procedures established by the Guide for the Care and Use of Laboratory Animals of the National Institutes of Health [19] and the precepts established by Law 11.794 of 8 October 2008 in conjunction with Decree 6.899 of 5 July 2009, which addresses the rules issued by the National Council for the Control of Animal Experimentation (CONCEA).

Adult male and female Swiss albino mice (*Mus musculus*) weighing between 25 and 35 g were utilized in this assay. The animals were separated into groups of four, based on weight or age, in micro-insulator cages (435 cm^2^) fitted with a valve to blow air in and out, grilles, and a lid, under conditions of 23 ± 1 °C and relative humidity between 40 and 60%. The animals were fed a diet of rodent-specific food from the Quintia brand, which had been sterilized by gamma radiation and provided ad libitum. The water was filtered through 5-micron activated charcoal and sterilized by autoclaving at 121 °C for 20 min, which was also provided ad libitum. Finally, the cages were lined with pine wood, which had also been sterilized by gamma radiation.

#### 2.7.2. Toxicological and Pharmacological Evaluation of *A. muricata* Extract

##### Acute Toxicity Assay on Mice

Two groups of three female animals were used for the acute oral toxicity test. The animals in the control group received the vehicle (50 μL of sterile buffered saline PBS), while the animals in the treated group received AMCE extract at a dose of 2000 mg/kg orally. Systematic behavioral observations were made at 15 and 30 min and 1, 2, and 4 h after administration, then daily until the fourteenth day using Hippocratic screening. Parameters such as body mass, water consumption, and feed consumption were observed every 24 h for 14 days. At the end of the experiment, the animals were weighed and anesthetized, then euthanized, and the organs (liver, spleen, heart, kidneys, and stomach) were removed, weighed, and macroscopically assessed.

##### Determination of the Therapeutic Dose Equivalent for Humans

The pharmacological potential of AMCE was evaluated using the zymosan-induced paw edema test according to the method of Anter et al. [20], with adaptations. For this purpose, groups of six male animals were organized. First, the animals in the treated group received 50 μL of a 2% zymosan solution in the plantar region of their right hind paws. In contrast, the contralateral (left) paw received 50 μL of sterile buffered saline (PBS), which served as a control. The treated groups were administered individual oral solutions containing 10, 100, and 200 mg/kg of AMCE, respectively. Furthermore, animals treated with the NSAID were utilized as a comparative positive control. At 30 min, 1 h, 2 h, 4 h, and 6 h, the volume of each mouse paw was measured in cubic millimeters (Δmm^3^) using a plethysmometer. The degree of paw swelling was quantified for each animal by comparing the observed change to the baseline control (treated with saline). The study was conducted in a blinded manner, with the individual responsible for the readings unaware of the identity of the animals in each group. The mice were identified by codes.

Finally, the extrapolation of the human equivalent dose (HED) was calculated using the method proposed by Nair and Jacob [21], adapted to the administration of AMCE (Equations (3) and (4)). In this sense, the dose for mice was determined considering the human conversion factor for albino mice according to body surface area. This was calculated by dividing it by the adult human weight of 70 kg and multiplying it by a factor to accommodate the animal’s body surface area. The exponent adopted for body surface area was a value of 0.67, which represents the difference in metabolic rate.

(3) $$\mathrm{HED}\;(\mathrm{mg}/\mathrm{kg})=({Animal}_{NOAEL}\;\mathrm{mg}/\mathrm{kg})\;\times \;\frac{{Animal}_{Weight}\;[kg]}{{{(Human}_{Weight}\;[kg])}^{\left(1-0.67\right)}\;}$$

(4) $$\mathrm{HED}\;(\mathrm{mg}/\mathrm{kg})=({Animal}_{DOSE}\;\mathrm{mg}/\mathrm{kg})\;\times \;\frac{{Animal}_{Km}}{{Human}_{Km}}$$

Considering that NOAEL is the dose where no adverse effects are observed, K_m_ is the correction factor.

#### 2.7.3. Evaluation of the Pharmacological Potential of U-PEO Matrices Containing *A. muricata* Concentrated Extract

In a similar manner to the aforementioned item, four groups of four male animals were established, with the plantar region of the left hind paw serving as the site for the assay. The first group received a volume of 50 μL of a 2% zymosan solution, while the second group was administered 50 μL of sterile buffered saline (PBS), serving as a negative control. In the treated group, 0.3 mL of an in situ film-forming solution containing a predetermined concentration of AMCE was administered topically. Similarly, the positive control group was treated with 0.3 mL of the *Symphytum officinale* herbal ointment (Section 2.1).

At pre-established time points of 30 min, 1 h, 2 h, 4 h, and 6 h, the volume of each paw was quantified using a plethysmometer both before and after edema stimulation. The degree of paw swelling was then determined for each animal, with the data expressed as the variation in edema volume (Δmm^3^).

### 2.8. Statistical Analysis

The results were expressed as the mean ± standard deviation of the triplicate. For linear regressions, a coefficient of determination close to 1 was considered (0.99 < R^2^ ≤ 1), and residuals were evaluated by one-way analysis of variance (ANOVA). The results of the in vivo tests were evaluated using GraphPad Prism^®^ software version 5.0 (GraphPad Software, San Diego, CA, USA). To determine the optimal therapeutic dose of AMCE, an analysis was conducted using ANOVA, followed by Tukey’s test. The Dunnett’s test was employed after ANOVA to evaluate the pharmacological activity of U-PEO loaded with AMCE. The statistical significance of the observed differences was determined using the following criteria: * *p* < 0.5, ** *p* < 0.1, or *** *p* < 0.01.

## 3. Results and Discussion

### 3.1. Evaluation of the Dried Leaf Powder and Total Flavonoid Content of A. muricata Concentrated Extract

The DLP quality control is presented in Table S1. The results of this analysis serve as important parameters for maintaining the homogeneity and reproducibility of the extraction processes involving AM leaves [22]. In this context, the powder obtained complies with the standards outlined in the Brazilian Pharmacopoeia, 6th edition, and is classified as moderately coarse, with a loss on drying of 8.82% (±3.4) and total ash of 8.04 ± 0.26 [14].

The extraction of chemical constituents from powders with different particle sizes is influenced by many factors, including the selective saturation of the solvent, the DLP concentration, and the technique selected [23]. This influence is particularly evident when low-energy methods are employed [24,25]. For this reason, the results demonstrated that maceration with 2% DLP in 70% hydroethanolic solvent was the optimal method for flavonoid extraction (Table 1), yielding 15.86 ± 1.80 mg of this secondary metabolite per gram of extract, approximately 46% higher than the other methods. The final mass of the concentrate obtained after rotaevaporation was 31.9 g, which corresponds to a yield of 31.9% in relation to the initial mass of the DLP used (100 g) for 5 L of the solvent used. This resulted in a final concentration of flavonoids, expressed as QE, of 39.27 mg ± 0.96 per gram of AMCE.

The high flavonoid content obtained in experiment 10 (Table 1) was the determining factor in selecting it for incorporation into U-PEO hybrid matrices and the subsequent in vitro and in vivo tests.

### 3.2. Antioxidant Activity and Cytotoxicity of the Leaf Extract of Annona muricata

The DPPH free radical scavenging test is a widely utilized method in the scientific literature for evaluating the antioxidant capacity of a given substance, thereby supporting the clarification of potential mechanisms associated with cell protection and viability [26]. In this regard, the outcomes of the test demonstrated that the samples exhibited a color change from purple to yellow (Supplementary Figure S3), with the intensity of the yellow hue directly proportional to the concentration of the extract. The DPPH free radical scavenging test resulted in samples that varied in color from yellow to purple (Supplementary Figure S3) on a scale directly proportional to the concentration of the extract. Consequently, the concentration of 50 µg/mL of AMCE was sufficient to inhibit 86.99% of DPPH, while the concentration of 5 µg/mL inhibited only 7.63%. Finally, the IC_50_ was calculated using the equation of the analytical curve generated (Supplementary Figure S4), resulting in a value of 29.18 µg/mL.

In their studies, Agu and Okolie [27] showed that if the aim is solely to explore the antioxidant activities of the different parts of AM, extractions with the solvent methanol-water or chloroform of the leaves or fruits yield superior results. This is attributed to the high content of flavonoids and phenols present in these portions. In contrast, when carrying out a similar test, Hasmila et al. [28] obtained a maximum antioxidant activity of 51.24% with 160 µg/mL, while in our studies, 86.99% of DPPH was captured only at 50 µg/mL, which demonstrates the influence of the high concentration of flavonoids in the sample.

**Figure 1:**
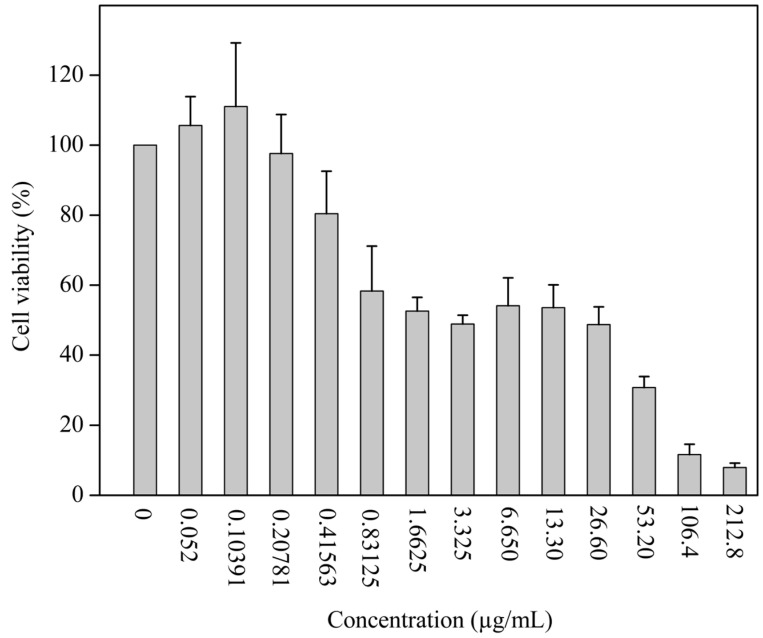
Cell viability (%) of HepG2 cells treated with different concentrations of *Annona muricata* concentrated extract. Results expressed as mean ± standard deviation, n = 3.

In turn, the MTT test is employed to ascertain the safety of a substance or ingredient against a specific population of human cells, thereby facilitating the determination of the median inhibitory concentration (IC50) for that particular strain [29]. Thus, the MTT test revealed that the IC_50_ of the concentrate was 8.95 ± 0.21 µg/mL, which corresponds to the dose of AMCE needed to eliminate 50% of the HepG2 cells tested. Figure 1 shows that concentrations equal to or less than 0.208 µg/mL present MTT of around 100%. The results of the lower concentrations with MTT above 100% also indicate the antioxidant activity of the compounds in this concentrate, which protects the cells from death [30]. Similar profiles of results can be found in the literature [31].

### 3.3. Synthesis of Ureasil–Polyether Hybrid Matrix

The U-PEO matrix is formed through the sol-gel process, a well-known method that allows for the incorporation of active ingredients with different polarities [10]. In brief, the PEO precursor contains approximately 12 oxyethylene units, which, when subjected to a hydrolysis and condensation reaction triggered by the addition of alcohol and water, favor interactions between the silanol groups and the consequent formation of a matrix network [9,16,32]. This network, in conjunction with other groups such as urea, is responsible for the high capacity for incorporating lipophilic and hydrophilic substances as well as metals [9,11,12]. Furthermore, the reaction time required for the formation of the firm gel phase is dependent on the equilibrium between the acid catalyst and the PEO employed.

**Figure 2:**
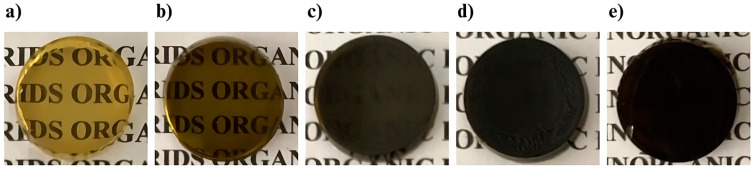
Visual aspects of U-PEO matrices containing AMCE 1% (**a**), 3% (**b**), 6% (**c**), 10% (**d**) and 20% (**e**). U-PEO: Ureasil–polyether hybrid matrix, AMCE: *Annona muricata* concentrated extract.

The optimal concentration of AMCE in the hybrid matrix was determined by incorporating varying proportions until the one that yielded an intact, homogeneous, and uniform material was reached. The color of the U-PEO exhibited a corresponding increase in intensity in proportion to the concentration of the extract added. In this regard, Figure 2 illustrates that the U-PEO containing 1% *w*/*v* (Figure 2a) and 3% *w*/*v* (Figure 2b) of AMCE exhibited a lighter appearance relative to the concentrations of 6% *w*/*v* (Figure 2c) and 10% *w*/*v* (Figure 2d), the latter of which exhibited initial signs of precipitation. This factor led to the selection of the 6% *w*/*v* proportion as the most promising for subsequent tests. Finally, upon reaching a concentration of 20% *w*/*v* (Figure 2e), the U-PEO matrix exhibited cracking and other imperfections.

### 3.4. Physicochemical Characterization of Hybrid Matrices

#### 3.4.1. Thermogravimetry (TGA)

**Figure 3:**
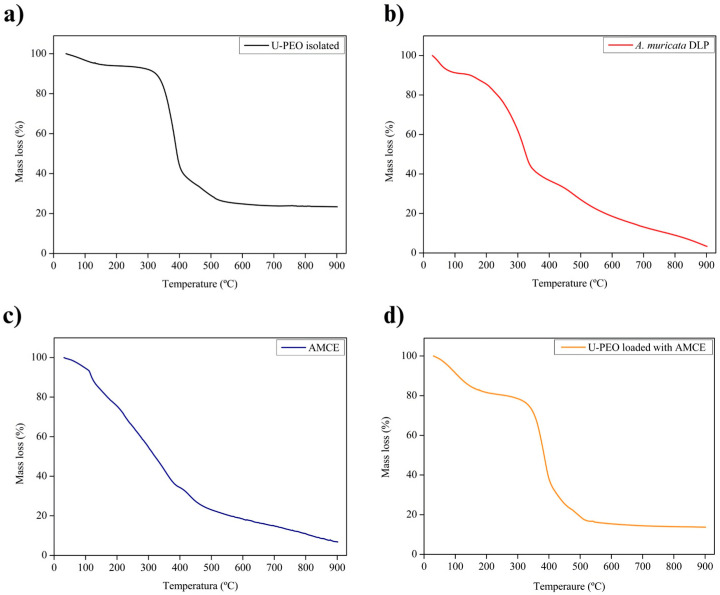
Thermogravimetric curves of the isolated U-PEO (**a**), dried leaf powder (**b**), *A. muricata* concentrated extract (**c**), and U-PEO loaded with *A. muricata* concentrated extract (**d**). U-PEO: Ureasil–polyether hybrid matrix, DLP: dried leaf powder, AMCE: *A. muricata* concentrated extract.

The thermal stability and physical compatibility of the ingredients were investigated by a TGA, which is based on the principle of mass loss under different temperature ranges, taking into account the endo- or exothermic energy balance of the matter or loss of volatiles [33]. Figure 3 shows the thermogravimetric curves of the DLP and AMCE as well as the U-PEO hybrid matrix isolated or loaded with AMCE. Table S3 shows the detailed TGA parameters.

The isolated U-PEO matrix showed three stages of significant mass loss (Figure 3a). The initial thermal event, which resulted in a loss of 8.03% of the total mass, occurred between 30 and 172.11 °C. This degradation was attributed to the evaporation of volatile solvents incorporated during synthesis, including water, absolute ethanol, and HCl [9,12]. The second event, which lasted until 421.75 °C, resulted in a loss of 58.94% of the total mass. This was primarily due to the degradation of the urea groups belonging to the polymer matrix, as previously reported in [9,12]. The third event lasted until 533.31 °C, with a loss of 13.80% of the mass due to the degradation of the siloxane bonds resulting from the synthesis reaction after the addition of the acid catalyst [9]. Finally, the final event, with no major loss of mass, occurred up to 900 °C, resulting in a loss of 3.27% and the degradation of the remaining organic traces, leaving a residue of 15.96%. This reflects the incomplete degradation of the hybrid material in the inert atmosphere of N_2_ [9,12].

In the case of the DLP and AMCE, both exhibited four thermal events (Figure 3b,c). The first thermal event occurred at approximately 31.53 to 134.31 °C, resulting in a mass loss of 9.10% for the DLP and 13.64% for AMCE. This loss can be attributed to the decomposition of volatile products, humidity, and the degradation of secondary metabolites, such as anthocyanins, polyphenols, and flavonoids [34]. The second event occurred between 134.31 and 368.57 °C, resulting in a mass loss of 50.30% (DPL) and 46.93% (AMCE). The third event occurred between 368.57 and 494.83 °C, resulting in a total loss of 11.94% (DLP) and 15.42% (AMCE). Both events suggest the degradation of macro- and micronutrients, including organic compounds [34,35]. The final event occurred at a temperature of up to 900 °C, resulting in a mass loss of 23.96% for the DLP and 16.39% for AMCE. This resulted in a residue of 4.70% for the DLP and 7.61% for AMCE, demonstrating incomplete degradation under a flow of N_2_.

Finally, the thermal degradation of the hybrid matrix loaded with AMCE (Figure 3d) exhibited a decomposition profile analogous to that of isolated U-PEO. Consequently, the AMCE decomposition occurred simultaneously with the loss of U-PEO matrix mass, which is indicative of the proper solubilization of AMCE. The absence of other events or a reduction in the temperature ranges corresponding to the degradation of the extract is indicative of physical compatibility. This phenomenon is also observed in the binary mixture between PEO and AMCE (Figure S5).

#### 3.4.2. Atomic Force Microscopy (AFM)

**Figure 4:**
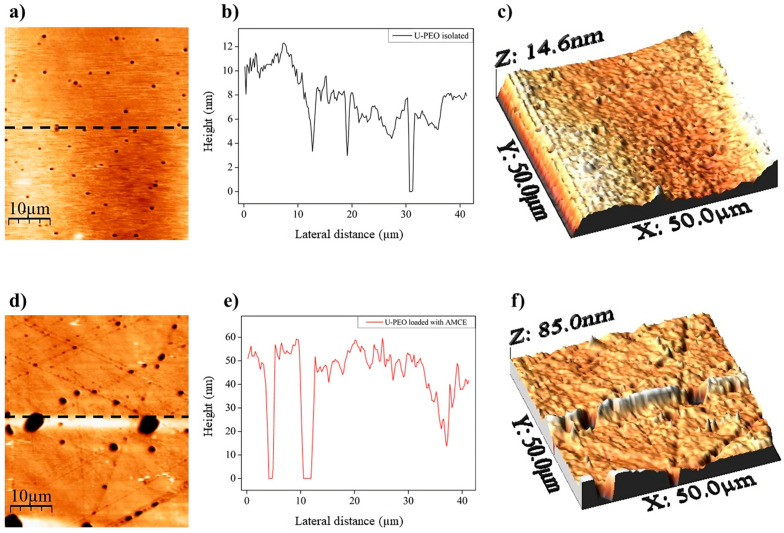
AFM topographic images of the U-PEO matrix isolated (**a**) or loaded with *A. muricata* concentrated extract (**d**). Surface profiles by height contour along the black line (**b**,**e**) of a and d, respectively. Three-dimensional representations (**c**,**f**) of a and d, respectively.

AFM is typically employed to examine the surface topography, facilitating the identification of features such as pores, roughness, and other variations that can influence parameters such as adhesion strength or drug release [36]. Figure 4 depicts the topographic surface of isolated U-PEO (Figure 4a) and loaded with AMCE (Figure 4d). The surface of the pure matrix is relatively smooth (Figure 4b), with variations in a maximum surface height of 14.60 nm (Figure 4c) and average of approximately 7.55 nm (Figure S6a), with an average roughness (Ra) of 1.90 nm and the presence of pores (average ≈ 393.75 nm in diameter), consistent with the literature [17]. However, when the extract was incorporated, the topographic surface exhibited greater roughness than that of the isolated U-PEO (Figure 4e), with an increase in the root mean square roughness (Ra) to 6.89 nm and a maximum height of 85 nm (Figure 4f), with variations of approximately 43.76 nm (Figure S6b). These parameters increased the pore diameter, with an average of approximately 928.80 nm.

One of the most significant factors contributing to the release profile of drugs is the increase in pores. This phenomenon directly affects the permeability of materials, allowing for greater fluid flow and improved diffusion, even in hydrophobic matrices [37]. In their study, Mikolaszek et al. [38] highlighted the significance of the porosity–permeability relationship for transdermal therapeutic systems. They posited that hollow pores could facilitate a greater penetration of the solvent into the material, thereby enabling the enhanced diffusion of the active ingredient into the medium. Furthermore, the increased surface roughness is also a relevant factor, as it favors the increased release of the incorporated active ingredient, allowing for a greater contact surface with the fluid [39].

#### 3.4.3. Macroscopic Swelling

**Figure 5:**
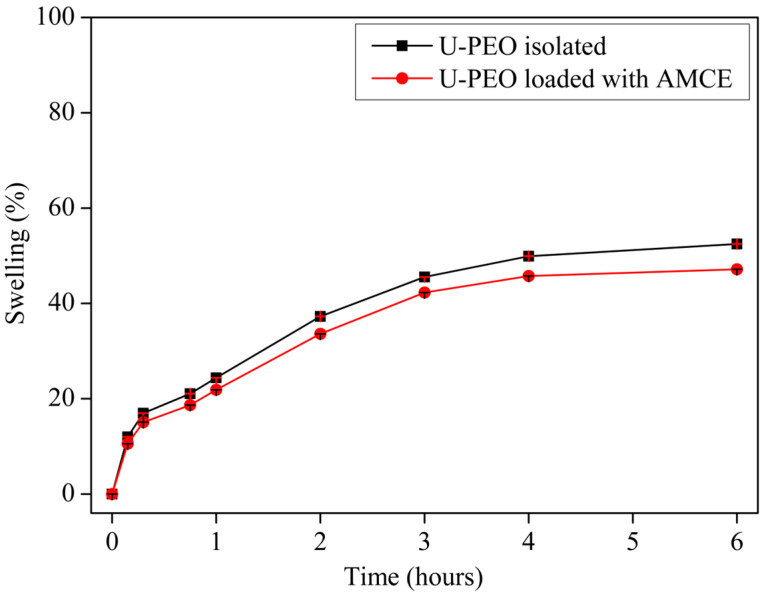
Swelling profile of U-PEO isolated (black curve) and loaded with concentrated *Annona muricata* extract (red curve). Results expressed as mean ± standard deviation, n = 3.

The swelling degree of U-PEO predicts its ability to interact with the receiving medium, considering the influence of different parameters, such as the pH and temperature of the fluid, as well as the type of polymer and the degree of cross-linking of the material. This may explain certain behaviors, such as the release profile of the incorporated active ingredient [40,41]. Figure 5 illustrates that U-PEO (black curve) retained its swelling profile even when loaded with AMCE (red curve). Both materials exhibited initial hydration of over 10% within the first 15 min, reaching a plateau within the first 6 h, with the isolated material swelling by 52.49% and the material incorporated with the extract swelling by 47.17%.

The swelling profile of U-PEO is directly related to its structure, considering the presence of hydrophilic groups, such as urea, ether-type oxygens, and lateral silanols, which are also responsible for a large part of the chemical interactions that allow for the incorporation of different pharmaceutical ingredients [42,43]. Other studies have also emphasized that when immersed in an aqueous medium, the distance between the siloxane nanodomains belonging to the organic–inorganic network increases due to the movement of the flexible polymer segments in such a way as to proportionally favor the macroscopic swelling of these materials [42,43,44].

As for AMCE, its hydrophilic character is related to the chemical structure of the components present in greater concentration in its portion. In this sense, the use of polar solvents in the extraction process, such as methanol or ethanol, has an affinity for obtaining phenolic compounds rich in hydroxyls, such as some quercetin derivatives, making the concentrate more hydrophilic [45]. Conversely, the study by Moraes et al. [46] also points out that when increasing the ethanol content, it is also able to recover lipophilic proteins; however, they tend to denature and precipitate in the same medium [46].

### 3.5. In Vitro A. muricata Release Assay

**Figure 6:**
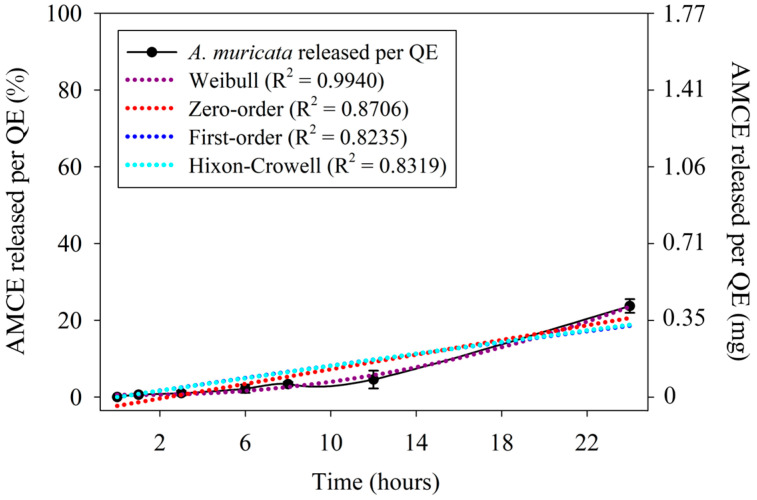
Release profile of *Annona muricata* concentrated extract per quercetin equivalent in different mathematical models. Results expressed as mean ± standard deviation, n = 6. AMCE: *Annona muricata* concentrated extract, QE: quercetin equivalent.

As displayed in Figure 6, the outcomes demonstrated the absence of a burst effect. The initial release of AMCE reached approximately 4.58% (0.081 mg of AMCE per QE) within the first 12 h, and a total cumulative release of about 23.72% (0.419 mg of AMCE per QE) was reached in 24 h, at which point it was observed that the release rate had reached a plateau. The burst effect is characterized by the immediate release of an active ingredient incorporated into a pharmaceutical form within the first few immersion times, suggesting an increase in the diffusion rate [47,48,49]. Although it is typical of various systems proposing modified or controlled release, avoiding the burst effect is necessary to provide greater predictability of the initial dose of the therapeutic agent as well as to avoid possible risks of initial toxicity [48,49].

Different models can be used as a basis for establishing a release profile predicting the standard behavior for the release of the drug from the matrix [17]. Those with the adjusted R^2^ closest to 1 were deemed optimal for analysis, as they presented a superior fit. In this context, zero-order release systems are capable of maintaining a constant dose of the incorporated active regardless of the concentration and lifetime of the device [48], whereas the first-order model predicts a similar release rate dependent on the concentration of the active [48,50]. In contrast, the model developed by Weibull correlates the logarithm of the active release with the logarithm of time, allowing for an interpretation related to the conformation of the system [51,52]. The studies by Hixson and Crowell reveal that the regularity of a surface is proportional to the cube root of its volume, attributing the release to geometric adjustment factors, such as dissolution, and not to a dependence on diffusion like the first two models mentioned above [52,53].

Among the expressions discussed, the kinetics developed by Weibull exhibited the greatest degree of fit (R^2^ = 0.9940) to the AMCE release profile recorded (Figure 6). For this model, the *b* coefficient suggests a predominant mechanism for the diffusion of the drug through the incorporated matrix. Consequently, values of *b* ≤ 0.75 indicate Fickian release in favor of fractal (*b* < 0.69) or Euclidean (0.69 < *b* < 0.75) spaces, while values between 0.75 < *b* < 1 suggest combinations between Fickian diffusion and Case II-type release [51,54]. In this context, the calculation for the coefficient yielded a value of *b* = 1.39, which is indicative of a sigmoid curve, a typical feature of complex mechanisms [51].

### 3.6. In Vivo Assays

#### 3.6.1. Acute Toxicity and Determination of the Therapeutic Dose of *A. muricata*

The results demonstrated that at a concentration of 2000 mg/kg, AMCE was not toxic to the treated animals and did not affect parameters such as feed and water consumption or organ weight, which were assessed over 14 days after euthanasia without any lesions appearing. Regarding the Hippocratic screening (Table S4), there were no significant changes in the majority of the observed behaviors. Nevertheless, between one and three hours, the animals exhibited drowsiness and drooping eyelids in comparison to the control group, which suggests a potential depressant effect of the extract [55]. These depressive symptoms were no longer observed after four hours of testing.

**Figure 7:**
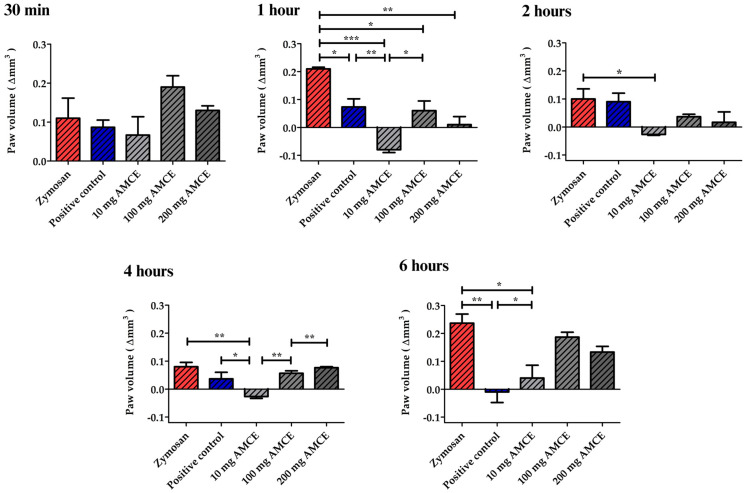
*Annona muricata* concentrated extract anti-inflammatory activity at different doses (10, 100, and 200 mg) evaluated by inhibiting zymosan-induced paw edema. * *p* < 0.5, ** *p* < 0.1, or *** *p* < 0.01. Results expressed as mean ± standard deviation, n = 6. AMCE: Annona muricata concentrated extract.

In turn, the therapeutic dose was determined using the paw edema test, which is based on the reduction of inflammatory activity in an animal model under the influence of an inflammatory agent (zymosan) [56]. As illustrated in Figure 7, at 30 min, there was no statistically significant difference between the negative control and the other treatments with the NSAID or AMCE. Surprisingly, it was observed that treatment with 10 mg/kg of AMCE was able to completely prevent the zymosan-induced edema between 1 and 6 h, maintaining significantly lower paw volume variation values throughout this period (* *p* < 0.5, ANOVA and Tukey’s test). While the positive control group treated with the NSAID demonstrated a reduction in inflammation between one and four hours, the inhibition of inflammation was only complete after six hours (** *p* < 0.1, ANOVA and Tukey’s test).

The higher doses of 100 mg/kg and 200 mg/kg demonstrated a significant reduction in the zymosan-induced edema in the first hour, with the effect sustained for two hours. However, the effect did not persist until the conclusion of the observation period. The inhibition percentages for the 4 and 6 h periods at the 100 mg/kg dose were 79.76 and 23.81%, respectively. In contrast, the inhibition percentages for the 4 and 6 h periods at the 200 mg/kg dose were 72.61 and 52.38%, respectively, when compared to their baseline. In light of the encouraging outcomes observed with the 10 mg/kg dose, the HED calculation proposed by Nair and Jacob [20] suggests that a 70 kg adult would require 43.05 mg of AMCE to elicit comparable anti-inflammatory effects, considering the discrepancy in body surface area between animals and humans (0.615 mg/kg).

The results demonstrate the influence of the high concentration of flavonoids present in AMCE. According to Tian et al. [57], anti-inflammatory activity is favored by the greater suppression of intracellular nitric oxide (NO) release caused by hydroxylated flavonoids or flavonoids with enol groups. These secondary metabolites were described by Manrique-de-la-Cuba et al. [58] as also being present in the leaves of A. muricata, with a particular emphasis on quercetin, catechin, epicatechin, and gallocatechin [58].

#### 3.6.2. Evaluation of the Pharmacological Potential of U-PEO Matrices

**Figure 8:**
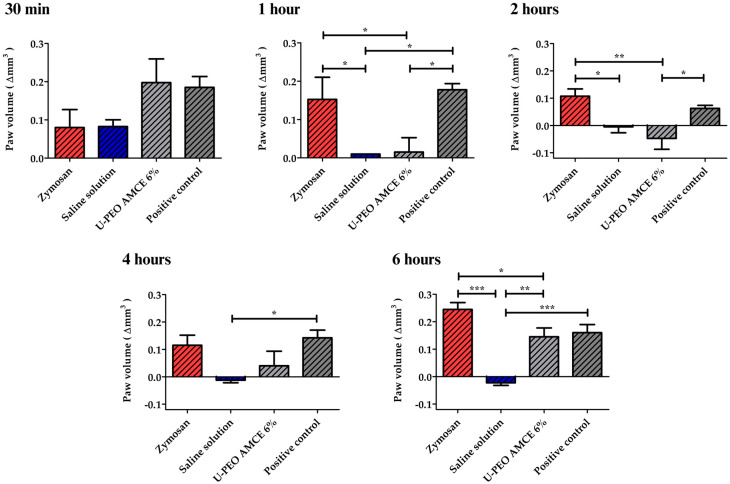
The anti-inflammatory activity of the U-PEO hybrid material loaded with *Annona muricata* concentrated extract was evaluated by inhibiting the zymosan-induced paw edema. * *p* < 0.5, ** *p* < 0.1, or *** *p* < 0.01. Results expressed as mean ± standard deviation, n = 4. AMCE: *Annona* muricata concentrated extract, U-PEO AMCE 6%: ureasil–polyether hybrid matrix loaded with 6% *w*/*v A. muricata* concentrated extract.

Figure 8 indicates that initially, there was no statistically significant difference between the negative control (treated with saline) and the other groups treated with the inflammatory agent zymosan, the film former containing AMCE (U-PEO AMCE 6%), and the positive control, treated with the herbal ointment (* *p* > 0.05, ANOVA and Tukey’s test). As expected, even after 30 min, the saline group showed no change throughout the treatment. However, after one hour, the zymosan group exhibited a significant increase in the inflammatory response, with volumes ranging from 0.36 to 0.52 mL, with the greatest values observed between four and six hours. This resulted in a variation in paw volume (Δmm^3^) of up to 0.27 mL.

As for the positive control group, at 30 min, 1 h, 2 h, 4 h, and 6 h, there was a reduction in the edema of 33.93%, 36.60%, 77.68%, 49.11%, and 42.86%, respectively, when compared to the baseline. The herbal medicine has anti-inflammatory activity closely linked to its secondary metabolites, such as allantoins, mucopolysaccharides, and tannins [59]. In comparison, the group treated with the in situ film-forming solution of the hybrid matrix loaded with 6% of AMCE demonstrated reductions in inflammation of 23.30%, 94.17%, 118.44%, 84.46%, and 43.68% at the respective times of 30 min, 1 h, 2 h, 4 h, and 6 h. The first peak in inflammation was observed in the first hour of treatment, with the inflammation remaining considerably suppressed for the following 4 h, resulting in a percentage close to 50% inhibition by the end of treatment. The results demonstrated superior outcomes when compared to the positive control group, which highlights the potential therapeutic benefits of the hybrid matrix developed.

Different pieces of the literature attribute the anti-inflammatory activity of AMCE to the hydroxylated flavonoids present in different regions of the plant, especially quercetin, catechin, and gallocatechin, which are also responsible for the greater suppression and release of intracellular nitric oxide (NO) or the inhibition of pro-inflammatory factors, such as tumor necrosis factor-alpha (TNF-α) [57,58,60]. For this reason, the anti-inflammatory therapeutic potential of the high concentration of flavonoids present in AMCE applied to a hybrid matrix, in comparison to other commercial formulations, suggests the potential for the development of more complex transdermal therapeutic systems, such as systems based on micro-needles, which allow for the therapeutic ingredient to pass through by overcoming barriers such as the molecular weight and hydrophilic profile of the incorporated substance [61]. Furthermore, they could facilitate the delivery of therapeutic agents through the stratum corneum without causing adverse effects, such as pain, irritation, or infection [61].

## 4. Conclusions

Our study presents a significant advancement in the development of novel transdermal therapeutic systems for the treatment of acute and chronic inflammation. The creation of a new polymeric matrix loaded with the Active Plant Pharmaceutical Ingredient from the concentrated extract of dried *Annona muricata* leaves, which contains a high concentration of flavonoids, represents a pivotal step in this direction. The matrix systems were produced from a urea-silicone polyether base using the sol-gel process and low-energy application. Preclinical tests of the *A. muricata* concentrated extract demonstrated the satisfactory suppression of zymosan-induced inflammation over a 6 h observation period. This indicates that lower doses of the concentrated extract may be employed to achieve therapeutic efficacy with a reduced risk of adverse effects. Consequently, the U-PEO hybrid matrix demonstrated modified release, prolonging the concentration of the active ingredient trapped within the system. This suggests a lower frequency of matrix replacement, enhancing patient adherence to treatment. Furthermore, the in vivo trials demonstrated the satisfactory suppression of inflammation over a 6 h observation period in comparison to a commercial formulation. Finally, clinical studies are necessary to confirm the safety and efficacy of the treatment in humans, allowing for the advancement of research towards the development of an optimal transdermal system. This may include the use of micro-needles to facilitate the passage of substances through the skin, overcoming barriers such as the stratum corneum or the hydrophilic profile and molecular weight of the substance while avoiding adverse effects, such as pain, irritation, or infection.

## Figures and Tables

**Table 1:** The concentration of total flavonoids per quercetin equivalent (mg per g of sample) of extracts obtained by different methods. Results expressed as mean ± standard deviation, n = 3.

Experiment	Method	Solvent	Dried Leaf Powder *	Total Flavonoids Expressed in Quercetin Equivalent
No.		Ethanol/Water *v*/*v*	% *w*/*v*	mg/g Extract ± SD **
01	Ultrasound	30:70	6	6.55 ± 1.49
02	Ultrasound	50:50	2	9.20 ± 1.36
03	Ultrasound	50:50	10	3.92 ± 0.38
04	Ultrasound	70:30	6	8.35 ± 0.49
05	Turbolysis	30:70	6	3.39 ± 0.44
06	Turbolysis	50:50	2	8.31 ± 0.11
07	Turbolysis	50:50	10	2.19 ± 0.70
08	Turbolysis	70:30	6	4.10 ± 0.26
09	Maceration	30:70	2	6.85 ± 0.43
10	Maceration	70:30	2	15.86 ± 1.80
11	Maceration	30:70	10	3.33 ± 0.42
12	Maceration	70:30	10	6.83 ± 0.21

SD: Standard deviation. * Mass of dried *A. muricata* leaf powder (for details of the particle size distribution, please refer to Table S2) per 100 g *w*/*v* of extractive solution. ** Mean ± Standard deviation (n = 3)

## Data Availability

The data presented in this study are available in this article and the Supplementary Materials.

## Supplementary Materials

The following supporting information can be downloaded at https://www.mdpi.com/article/10.3390/pharmaceutics16081097/s1, Table S1: Quality control of powder obtained from dried *A. muricata* leaves; Table S2: Particle size distribution of dried *A. muricata* leaf powder; Table S3: TGA parameters of isolated samples and U-PEO loaded with *A. muricata* concentrated extract; Table S4: Signs of toxicity of *Annona muricata* concentrated extract observed through Hippocratic screening; Figure S1: Obtaining the powder from the *A. muricata* dried leaves. Collection and weighing (a), the distribution in trays (b), drying in a forced-air oven (c,d), and pulverization in a 10-mesh knife mill (e,f); Figure S2: Linear regression (a) and residual graph (b) of *A. muricata* concentrated extract per quercetin equivalent; Figure S3: Distinction of colors and shades of DPPH solutions with different concentrations of *Annona muricata* hydroalcoholic extract. Distinction of colors and shades of DPPH solutions with different concentrations of *Annona muricata* concentrated extract. (a) DPPH 0.20 mM + methanol (3 mL 1:1 *v*/*v*); (b) AMCE 5 µg/mL + DPPH 0.20 mM (3 mL 1:1 *v*/*v*); (c) AMCE 10 µg/mL + DPPH 0.20 mM (3 mL 1:1 *v*/*v*); (d) AMCE 20 µg/mL + DPPH 0.20 mM (3 mL 1:1 *v*/*v*); (e) AMCE 30 µg/mL + DPPH 0.20 mM (3 mL 1:1 *v*/*v*); (f) AMCE 40 µg/mL + DPPH 0.20 mM (3 mL 1:1 *v*/*v*); (g) AMCE 50 µg/mL + DPPH 0.20 mM (3 mL 1:1 *v*/*v*); and (h) AMCE 100 µg/mL + DPPH 0.20 mM (3 mL 1:1 *v*/*v*); Figure S4: Linear regression of DPPH free radical scavenging content (a) and residual plot (b) versus *A. muricata* extract concentration; Figure S5: Thermogravimetric curves of the PEO hybrid precursor (black curve), *A. muricata* concentrated extract (red curve), and the 1:1 binary combination (blue curve); Figure S6: The average height of the topographic surface of U-PEO isolated (a) or loaded with *A. muricata* concentrated extract (b).
