# Supplementary material for: The Development and Pre-Clinical Anti-Inflammatory Efficacy of a New Transdermal Ureasil–Polyether Hybrid Matrix Loaded with Flavonoid-Rich Annona muricata Leaf Extract
Source: Pharmaceutics. 2024 Aug 21;16(8):1097. doi: 10.3390/pharmaceutics16081097 (PMC11359889; doi:10.3390/pharmaceutics16081097)
Supplement: Supplementary file 1 [file pharmaceutics-16-01097-s001.zip › pharmaceutics-3090319-supplementary.pdf]

pharmaceutics

MDPI

Development and Pre-Clinical Anti-Inflammatory Efficacy of a New Transdermal Ureasil-Polyether Hybrid Matrix Loaded with Flavonoid-Rich *Annona muricata* Leaf Extract

Camila Beatriz Barros Araújo, José de Oliveira Alves-Júnior, Mariana Rillo Sato, Kammila Martins Nicolau Costa, Jéssica Roberta Lima, Bolívar Ponciano Goulart de Lima Damasceno, José Batista de Lima Junior, Bruna Galdorfini Chiari Andréo, Vanda Lúcia dos Santos and João Augusto Oshiro-Junior

SUPPLEMENTARY TABLES

Table S1. Quality control of powder obtained from dried *A. muricata* leaves

Tests		Results obtained
Particle size analysis	-	Moderately coarse powder
Apparent density	g/mL	0.30 ± 0.01
Loss due to desiccation	%	8.82 ± 3.40
Determining pH	-	5.54 ± 0.11
Total ash	%	8.04 ± 0.26
Acid-insoluble ash	%	1.56 ± 0.19

Table S2. Particle size distribution of dried *A. muricata* leaf powder

Sieve mesh opening size (µm)	Retained mass (g)	Retained mass (%)
**710**	0.57	2.30
**355**	3.50	14.30
**180**	6.70	27.40
**150**	0.99	4.05
**75**	9.02	36.90
**38**	3.13	12.82
**Collector**	0.53	2.17

Table S3. TGA parameters of isolated samples and U-PEO loaded with *A. muricata* concentrated extract

Sample	Step 1		Step 2		Step 3		Step 4		Residue
	T_{onset} - T_{endset} (°C)	Δm (%)	T_{onset} - T_{endset} (°C)	Δm (%)	T_{onset} - T_{endset} (°C)	Δm (%)	T_{onset} - T_{endset} (°C)	Δm (%)	(%)
U-PEO isolated	30.00 - 172.11	8.03	172.11 - 421.75	58.94	421.75 - 533.31	13.80	421.75 - 900.00	3.27	15.96
A. muricata DLP	31.53 - 134.31	9.10	134.31 - 368.57	50.30	368.57 - 494.83	11.94	494.83 – 900.00	23.96	4.70
AMCE	31.91 - 106.47	6.12	106.47 - 227.11	23.60	227.11 - 402.89	35.76	402.89 - 900.00	7.48	7.48
U-PEO + AMCE*	29.46 - 193.33	18.01	193.33 - 427.37	51.71	427.37 - 550.05	13.67	550.05 - 900.00	2.54	14.07

* Sample shown in Figure S4

Caption. U-PEO: Ureasil-polyether hybrid matrix. DLP: dried leaf powder. AMCE: *A. muricata* concentrated extract

Table S4. Signs of toxicity of *Annona muricata* concentrated extract observed through hippocratic screening

**Parameters**	**Time**									
**CNS**	**30 min**		**1 hour**		**2 hours**		**3 hours**		**4 hours**	
	**C**	**T**	**C**	**T**	**C**	**T**	**C**	**T**	**C**	**T**
**1. Stimulating**										
Agitation	0	0	0	0	0	0	0	0	0	0
Aggressiveness	0	0	0	0	0	0	0	0	0	0
Increased ambulation	0	0	0	0	0	0	0	0	0	0
Walking in circles	0	0	0	0	0	0	0	0	0	0
Self-cleaning	0	1	0	0	0	0	0	0	0	0
Yawning	0	0	0	0	0	0	0	0	0	0
Abdominal twitching	0	0	0	0	0	0	0	0	0	0
Convulsions	0	0	0	0	0	0	0	0	0	0
Climbing	0	0	0	0	0	0	0	0	0	0
Vocal fremitus	0	0	0	0	0	0	0	0	0	0
Irritability	0	0	0	0	0	0	0	0	0	0
Standing up	1	1	0	0	0	0	1	0	0	0
Stereotyped movement	0	0	0	0	0	0	0	0	0	0
Vibrissae movement	0	0	0	0	0	0	0	0	0	0
Pedaling	0	0	0	0	0	0	0	0	0	0
Response to touch	3	3	3	3	3	3	3	3	3	3

Shaking the head	0	0	0	0	0	0	0	0	0	0
Jumping	0	0	0	0	0	0	0	0	0	0
Salivation	0	0	0	0	0	0	0	0	0	0
Tachycardia	0	0	0	0	0	0	0	0	0	0
Tremors	0	0	0	0	0	0	0	0	0	0
Vocalization	0	0	0	0	0	0	0	0	0	0
Reflux	0	0	0	0	0	0	0	0	0	0
**2. Depressant**										
Hind leg abduction	0	0	0	0	0	0	0	0	0	0
Decreased ambulation	0	0	0	0	0	0	0	0	0	0
Analgesia	0	0	0	3	0	3	0	0	0	0
Anesthesia	0	0	0	0	0	0	0	0	0	0
Ataxia	0	0	0	0	0	0	0	0	0	0
Catatonia	0	0	0	0	0	0	0	0	0	0
Stretching	0	0	0	0	0	0	0	0	0	0
Hypnosis	0	0	0	0	0	0	0	0	0	0
Loss of ear reflex	0	0	0	0	0	0	0	0	0	0
Loss of corneal reflex	0	0	0	0	0	0	0	0	0	0
Eyelid ptosis	0	0	0	0	0	0	0	0	0	0
Sedation	0	0	0	0	0	0	0	0	0	0
Drowsiness	0	0	0	1	0	3	0	3	0	3

Caption. CNS (Central Nervous System); C (Saline Control); T (Treated control). Classification of effects: (0) no effect; (1) low effect; (2) medium effect; (3) intense effect.

SUPPLEMENTARY FIGURES

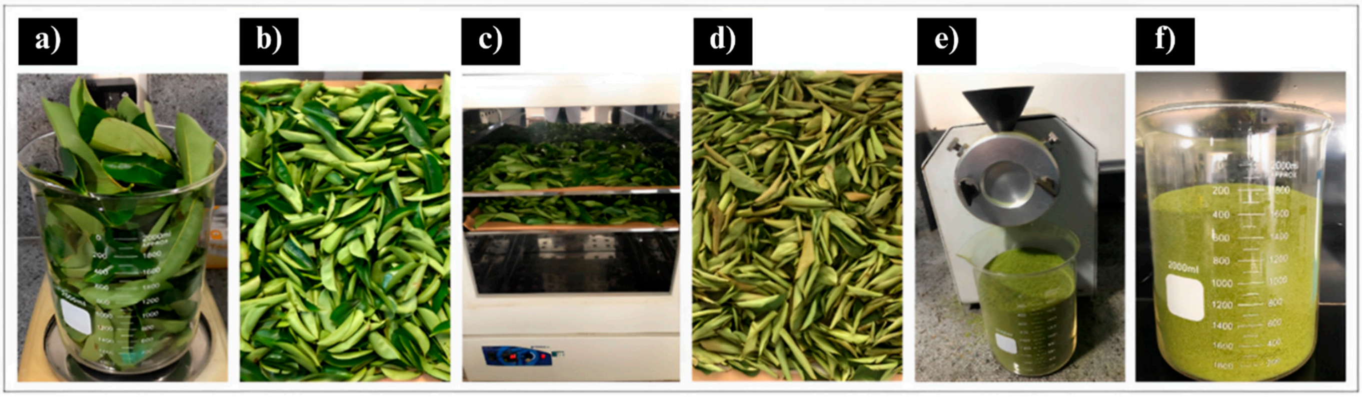

Figure S1. Obtaining the powder from the *A. muricata* dried leaves. Collection and weighing (a), distribution in trays (b), drying in a forced-air oven (c and d), and pulverization in a 10-mesh knife mill (e and f)

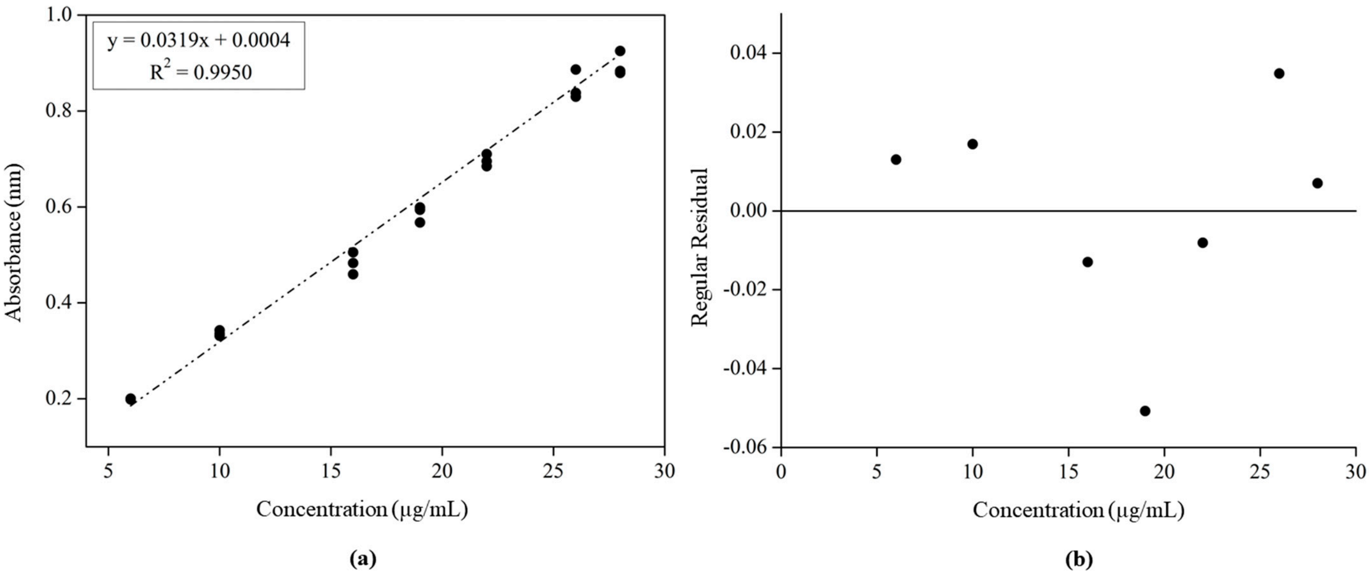

Figure S2. Linear regression (a) and residual graph (b) of *A. muricata* concentrated extract per quercetin equivalent

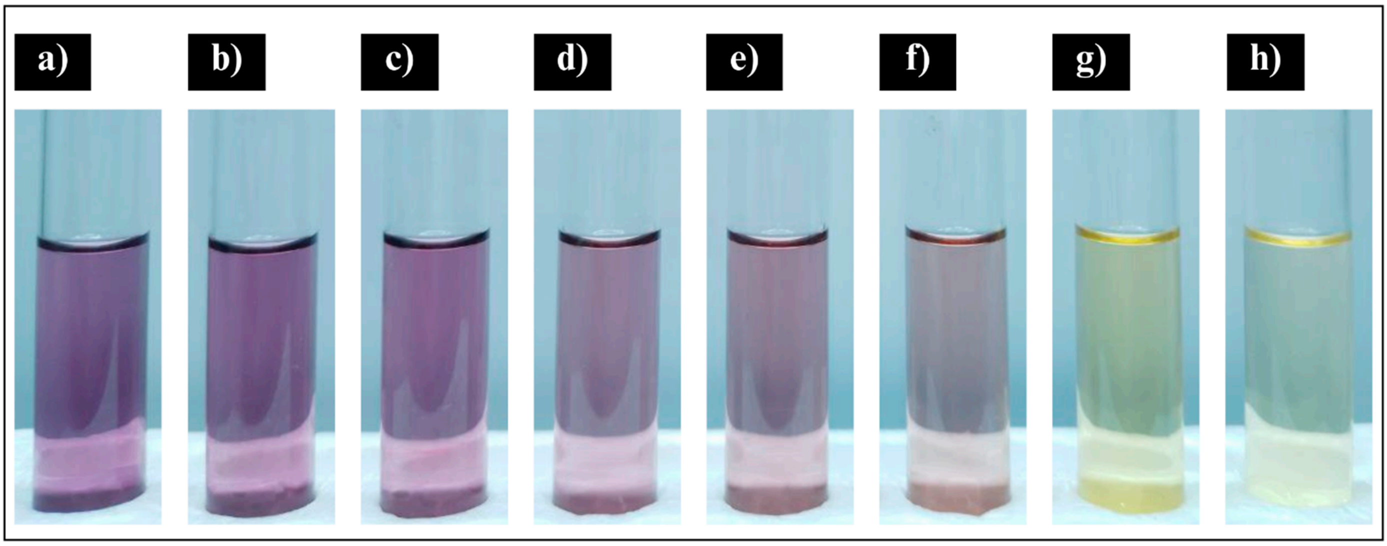

Figure S3. Distinction of colors and shades of DPPH solutions with different concentrations of *Annona muricata* hydroalcoholic extract. Distinction of colors and shades of DPPH solutions with different concentrations of *Annona muricata* concentrated extract. a) DPPH 0.20 mM + Methanol (3 mL 1:1 *v/v*); b) AMCE 5 μg/mL + DPPH 0.20 mM (3 mL 1:1 *v/v*); c) AMCE 10 μg/mL + DPPH 0.20 mM (3 mL 1:1 *v/v*); d) AMCE 20 μg/mL + DPPH 0.20 mM (3 mL 1:1 *v/v*); e) AMCE 30 μg/mL + DPPH 0.20 mM (3 mL 1:1 *v/v*); f) AMCE 40 μg/mL + DPPH 0.20 mM (3 mL 1:1 *v/v*); g) AMCE 50 μg/mL + DPPH 0.20 mM (3 mL 1:1 *v/v*) and h) AMCE 100 μg/mL + DPPH 0.20 mM (3 mL 1:1 *v/v*)

Caption. AMCE: *Annona muricata* concentrated extract.

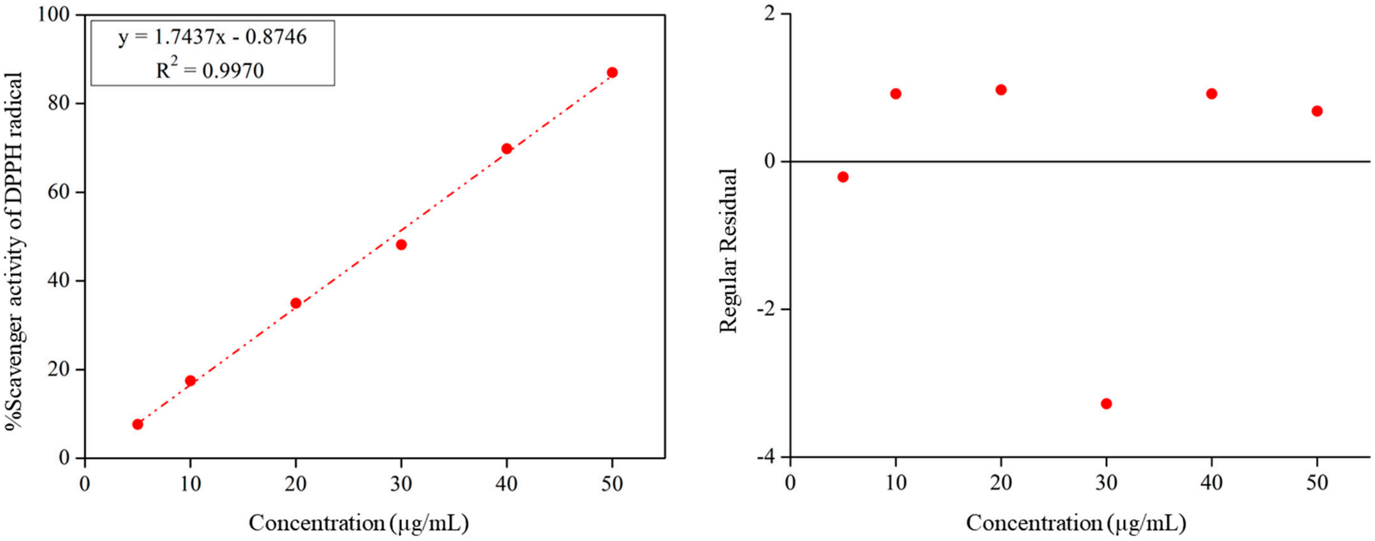

(a) **(b)**

Figure S4. Linear regression of DPPH free radical scavenging content (a) and residual plot (b) versus *A. muricata* extract concentration

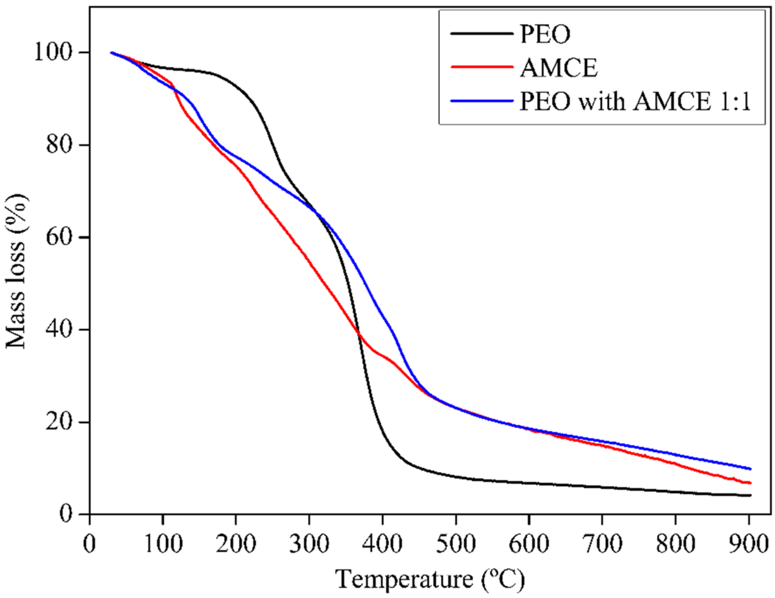

Figure S5. Thermogravimetric curves of the PEO hybrid precursor (black curve), *A. muricata* concentrated extract (red curve) and the 1:1 binary combination (blue curve)

Caption. PEO: Hybrid precursor; AMCE: *Annona muricata* concentrated extract.

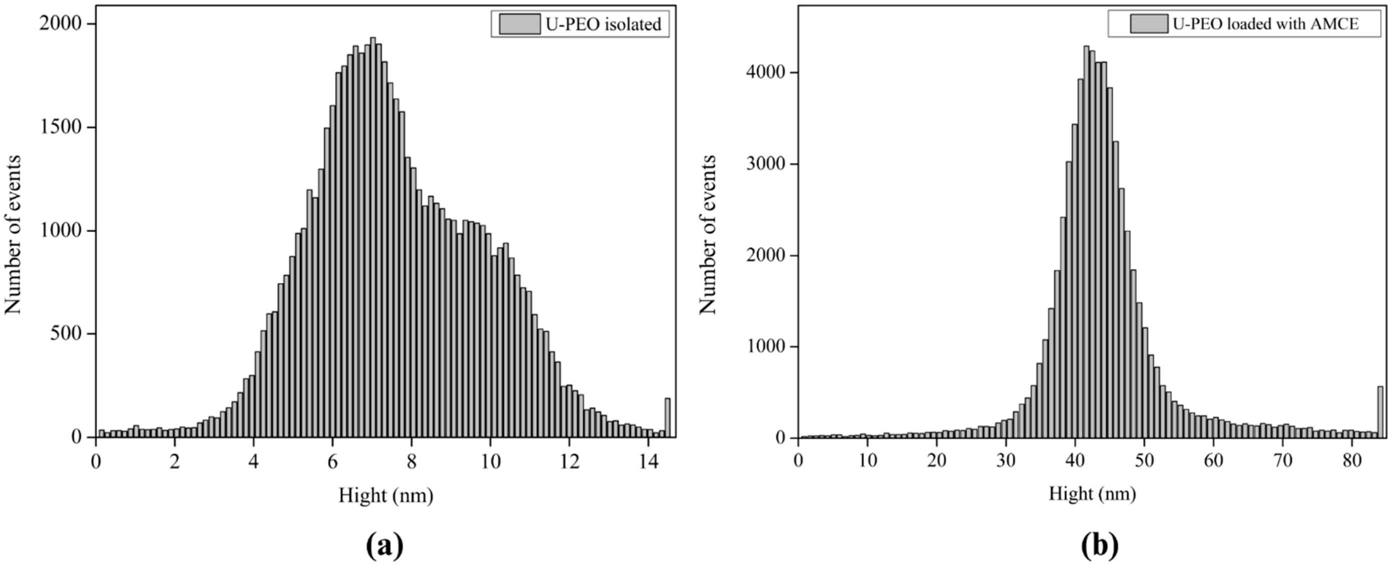

Figure S6. Average height of the topographic surface of U-PEO isolated (a) or loaded with *A. muricata* concentrated extract (b)
Caption. U-PEO: Ureasil-polyether hybrid, AMCE: *Annona muricata* concentrated extract.
